# DJC Suppresses Advanced Glycation End Products-Induced JAK-STAT Signaling and ROS in Mesangial Cells

**DOI:** 10.1155/2017/2942830

**Published:** 2017-05-29

**Authors:** Min Sun, Yan Li, Wenjie Bu, Jindong Zhao, Jianliang Zhu, Lingling Gu, Pingping Zhang, Zhaohui Fang

**Affiliations:** ^1^Anhui Province Key Laboratory of R&D of Chinese Medicine, School of Life Science, Anhui University, Hefei, Anhui 230601, China; ^2^Department of Endocrinology, The First Affiliated Hospital of Anhui University of TCM, Hefei, Anhui 230031, China

## Abstract

The antidiabetic properties and anti-inflammatory effects of Danzhi Jiangtang Capsules (DJC) have been demonstrated in clinical and laboratory experiments. In this study, we explored whether DJC can ameliorate advanced glycation end products- (AGEs-) mediated cell injury and the precise mechanisms of DJC in treating diabetic nephropathy (DN). Western blot analysis was employed to assess the expressions of iNOS, COX2, and SOCS and the phosphorylation of JAK2, STAT1, and STAT3 in glomerular mesangial cells (GMCs) after treatment with DJC. TNF-*α*, IL-6, and MCP-1 were determined using double-antibody sandwich ELISA. ROS and NADPH oxidase activity were measured by DCFH-DA assay and lucigenin-enhanced chemiluminescence, respectively. DJC significantly reversed the AGEs-induced expression of COX2 and iNOS. Moreover, DJC inhibited the AGEs-induced JAK2-STAT1/STAT3 activation, resulting in the inhibition of inflammatory cytokines such as IL-6, MCP-1, and TNF-*α* in a concentration-dependent manner. The ability of DJC to suppress STAT activation was also verified by the observation that DJC significantly increased the SOCS3 protein level. DJC reversed the AGEs-induced accumulation of ROS and NADPH oxidase activity, thus confirming that DJC possesses antioxidant activity. The results suggest that the anti-inflammatory effects of DJC in GMCs may be due to its ability to suppress the JAK2-STAT1/STAT3 cascades and reduce ROS production.

## 1. Introduction

Advanced glycation end products (AGEs), which are formed through a nonenzymatic reaction between reducing sugars and free amino groups of proteins, lipids, or nucleic acids, play an important role in the pathogenesis of diabetic nephropathy (DN). AGEs act via multiple mechanisms, such as oxidative stress generation and overproduction of various growth factors and cytokines, and are thus considered a key factor in renal structural and functional alterations, such as interstitial fibrosis, fibrotic glomeruli, tubular atrophy, and mesangial expansion [1].

The Janus kinase-signal transducers and activators of transcription (JAK-STAT) signaling pathway is an essential signaling pathway of inflammatory cytokines and is activated in DN [2]. The binding of cytokines to receptors may induce the phosphorylation of receptor-associated JAK, which in turn leads to the phosphorylation of STATs. Phosphorylated STATs are dissociated from the receptor complex and then form homodimers or heterodimers, and they then translocate into the nucleus to regulate the transcription of target genes encoding inflammatory cytokines such as IL-6, TNF-*α*, and MCP-1 and inducible enzymes including inducible nitric oxide synthase (iNOS) and cyclooxygenase 2 (COX2). Further, the JAK-STAT signaling pathway is negatively regulated by the suppressor of cytokine signaling (SOCS) proteins [3, 4]. The SOCS proteins are induced by many cytokines and pathogenic mediators and therefore act in a negative feedback loop to inhibit further signal transduction [3].

Chronic inflammation is a potential factor in the development and progression of DN [2]. JAK-STAT signal transduction pathway is activated by pathogenic mediators including angiotensin II (Ang II), AGEs, and high glucose in renal cells during DN [2, 5–7]. The formation of AGEs is accompanied by oxidative stress that, in turn, generates reactive oxygen species (ROS). Furthermore, the binding of AGEs to receptors for AGEs (RAGE) initiates ROS production via NADPH oxidase [8].

Danzhi Jiangtang Capsule (DJC) is a traditional Chinese medicine compound that has been used to treat diabetes and DN in the clinics for more than 10 years [9–11]. We previously demonstrated that DJC inhibits oxidative stress [10] and downregulates the high expression of inflammatory factors including IL-8, TNF-*α*, CXCL-5, CXCL-9, and MCP-1 [11–14]. DJC has also been shown to inhibit NF-*κ*B and ameliorates renal inflammation in diabetic rats and patients [11, 15]. Clinical reports have shown that DJC reduces urinary albumin excretion rate (UAER) in patients with early DN [10, 11]. However, it is still not known whether DJC acts via the JAK-STAT pathway. In this study, we hypothesized that DJC may improve renal function via a mechanism that involves JAK-STAT pathway activation and antioxidant activity. To test this hypothesis, we evaluated the antioxidant effects of DJC and investigated the effects of DJC on COX2 and iNOS protein levels and the levels of inflammatory cytokines. The roles of DJC in AGEs-induced JAK-STATs signaling and SOCS protein expression were also investigated.

## 2. Materials and Methods

### 2.1. Preparation of DJC

DJC was obtained from the Department of Pharmaceutics at the First Affiliated Hospital of Anhui University of Traditional Chinese Medicine (Hefei, Anhui, China). DJC contains 6 medicinal components: Radix Pseudostellariae, Radix Rehmanniae, Cortex Moutan, Rhizoma Alismatis, Semen Cuscutae Chinensis, and Hirudo, at a ratio of 6 : 5 : 4 : 4 : 3 : 3. The herbal drug was authenticated and standardized on marker compounds according to the Chinese Pharmacopoeia 2005 [16]. Briefly, Cortex Moutan was extracted by 95% ethanol, the extract was granulated, and the gruffs were mixed with the other 4 herbal materials to perform wet distillation. The water extract was precipitated with alcohol and then the supernatant was dried and crushed. The resulting powder was mixed with the Cortex Moutan granules and the Hirudo fine powder to prepare DJC [17]. To reduce the batch-to-batch variability, the species, origin, harvest time, medicinal parts, and concocted methods for each component were strictly standardized. Moreover, ultraperformance liquid chromatography (UPLC) was applied to quantitate the components of the DJC capsule [18].

### 2.2. Chemicals and Reagents

Low glucose Dulbecco's modified Eagle's medium (DMEM)/F12 was obtained from Life Technology (Carlsbad, CA, USA), and fetal bovine serum (FBS) was obtained from Sijiqing Biological Engineering Materials Co., Ltd. (Hangzhou, Zhejiang, China). Mouse IL-6, TNF-*α* ELISA kits were purchased from ExCell Bio (Shanghai, China), and MCP-1 kit was purchased from NeoBioscience (Shenzhen, Guangdong, China). The polyclone antibodies against p-JAK2 and JAK2 were obtained from Millipore (Billerica, MA, USA), and COX2, p-STAT1 (Tyr 701), and STAT1 polyclone antibodies were purchased from SAB (Signalway Antibody company, College Park, MD, USA). The polyclone antibodies against SOCS1 were from ImmunoWay Biotechnology (Plano, TX, USA). The polyclone antibodies against SOCS3 and iNOS were purchased from Santa Cruz Biotechnology (Santa Cruz, CA, USA). The monoclonal antibodies against p-STAT3 (Tyr 705) and STAT3 were purchased from Cell Signaling Technology (Beverly, MA, USA). Rabbit polyclonal *β*-actin antibody and all of the secondary antibodies used for western blotting were purchased from Abmart (Hangzhou, Zhejiang, China). Electrochemiluminescence (ECL) kit and BCA Protein Assay Kit were obtained from Thermo Scientific Pierce (Rockford, IL, USA). 3-(4,5-Dimethyl-2-thiazolyl)-2,5-diphenyl-2-H-tetrazolium bromide (MTT) and dichlorofluorescin diacetate (DCFH-DA) were obtained from Sigma (St. Louis, MO, USA). Lucigenin and NADPH were obtained from TCI (Tokyo, Japan) and Roche (Indianapolis, IN, USA), respectively. AG490 was purchased from Selleck Chemicals (Houston, TX, USA). Apocynin (APO) was from Alfa Aesar Chemical (Tianjin, China). All other chemicals were of analytical grade and were obtained commercially.

### 2.3. Cell Culture

Mouse glomerular mesangial cells (GMCs) were obtained from the Cell Bank of the Type Culture Collection of Chinese Academy of Sciences (Shanghai, China). The cells were cultured in low glucose DMEM/F12 medium supplemented with 10% FBS, 100 U/mL penicillin, 100 *μ*g/mL streptomycin, and 10 mM HEPES. The cell line was maintained in an incubator at saturated humidity at 37°C under 5% CO_2_. In this study, cells were cultured in DMEM/F12 containing 5% FBS, various concentrations of DJC (0.125, 0.5, and 2 mg/ml), or the JAK2 kinase inhibitor AG490 (20 *μ*M) for 48 h. DJC was dissolved in dimethyl sulfoxide (DMSO) to a final concentration of less than 0.1% in DMEM/F12. DJC at 0.125, 0.5, and 2 mg/ml were defined as low (L), medium (M), and high (H) concentrations, respectively. Each experimental data point represents the mean of duplicate wells from three independent experiments.

### 2.4. Preparation of AGEs

Briefly, bovine serum albumin (BSA) (40 mg/ml) was incubated with D-glucose (0.5 mM) in 0.15 M phosphate buffer at 37°C for 60 days. Preparations were lyophilized, resuspended in water, and dialyzed against phosphate buffered saline (PBS) to remove free glucose [19]. Control BSA was prepared by identical incubation without glucose. Glycation was assessed by characteristic fluorescence (excitation 370 nm, emission 440 nm) with a 9.7-fold increase in the fluorescence of AGEs compared to control BSA.

### 2.5. Measurement of Cytokines by ELISA

GMCs (1 × 10^4^ cells in 1 mL) were seeded on 24-well plates in triplicate and cultured with AGEs at a concentration of 250 *μ*g/ml for 48 h in the presence or absence of graded concentrations of DJC at 0.125, 0.5, and 2 mg/ml or AG490 (20 *μ*M). After stimulation, culture media were collected and centrifuged at 12,500 rpm for 5 min. The supernatants were collected for the measurement of IL-6, TNF-*α*, and MCP-1. Cytokines in the medium were determined by a quantitative sandwich enzyme-linked immunosorbent assay (ELISA) according to the manufacturer's instructions.

### 2.6. Western Blotting

GMCs were rinsed three times with ice-cold PBS and were then harvested and lysed in ice-cold RIPA buffer (50 mM Tris, pH 7.5, 150 mM NaCl, 2 mM EGTA, 2 mM Na_3_VO_4_, and 1 mM PMSF) containing HALT protease/phosphatase inhibitor cocktail (Sangon Biotech, Shanghai, China). The cell lysates were centrifuged at 4°C for 20 min at 14,000 rpm, and the protein concentration in the supernatants was detected using a Thermo Scientific Pierce BCA Protein Assay Kit. Aliquots of the lysates were separated using 8–10% SDS-PAGE and transferred to nitrocellulose membranes (Bio-Rad, Hercules, CA, USA). The membranes were blocked in PBST buffer containing nonfat milk for 1 h and probed with appropriate primary antibodies at 4°C overnight, washed, and incubated with secondary antibodies for 2 h at room temperature and finally were developed with the ECL detection reagents. The antibody-antigen complexes were visualized by the FluorChem™ E System (Protein Simple, San Jose, CA, USA), and the Image-Pro Plus software for densitometry analysis was used to quantify the protein levels.

### 2.7. Detection of ROS

GMCs (1 × 10^4^ in 200 *μ*L) were seeded in quadruplicate on a 96-well plate and incubated with AGEs at a concentration of 250 *μ*g/ml for 48 h in the presence or absence of various concentrations of DJC. Culture plates were washed three times with PBS (pH 7.4) and incubated in DMEM/F12 (phenol-red-free) containing DCFH-DA (10 *μ*M) at 37°C for 30 min. The cells were then washed with prewarmed PBS and covered with 100 *μ*l DMEM/F12 without phenol red, and the fluorescence intensity (FI) of each well was measured by a POLARstar OPTIMA Multidetection Microplate Reader (Isogen Life Science, De Meern, Netherlands) at Ex 485/Em 530 nm. The fluorescence levels were expressed as the percent of the control fluorescent level.

### 2.8. Measurement of NADPH Oxidase Activity

NADPH oxidase activity was measured by lucigenin-enhanced chemiluminescence as previously described [20]. GMCs (5 × 10^4^ cells in 2 mL) were seeded on 12-well plates in quadruplicate and cultured with AGEs (250 *μ*g/ml) for 48 h in the presence or absence of graded concentrations of DJC or AG490. After treatment, the cells were thoroughly washed by ice-cold PBS and resuspended in 500 *μ*l of ice-cold hypotonic lysis buffer of pH 7.4 (20 mM Tris-HCl, 1 mM EDTA, and 1 mM EGTA and protease inhibitors). The cell suspensions were sonicated and centrifuged at 15,000 rpm for 10 min. The resulting pellets were resuspended and sonicated in lysis buffer. The supernatants were then centrifuged at 15,000 rpm for 60 min. The pellets containing whole cell membranes were resuspended in assay buffer (10 mM sodium phosphate, 2 mM potassium phosphate, 10 mM potassium chloride, 50 mM triethanolamine, 150 mM NaCl, 2 mM MgCl_2_, 1 mM EDTA, and protease inhibitors) containing 10 *μ*M lucigenin and 100 *μ*M NADPH. The activity was measured using a Tecan Infinite 200 Multimode microplate reader (Männedorf, Switzerland). Values expressed as arbitrary light units (ALUs) per 100 *μ*g protein were converted to the percent change from control. All of the protein concentrations were assayed using a BCA Protein Assay Kit.

### 2.9. Evaluation of Cell Viability

Cell viability was determined using the MTT assay. Cells were seeded on 96-well plates 24 h before treatment. The cells were then treated with various concentrations of DJC for 48 h, and then 20 *μ*L MTT (5 mg/mL) was added to the culture medium in each well for 2–4 h until a purple precipitate was visible. Cells were then treated with 150 *μ*L DMSO to dissolve the crystals and left at room temperature in the dark for 2 hours. The absorbance at 490 nm was recorded using a Tecan Infinite 200 Multimode microplate reader (Männedorf, Switzerland). Data are presented as a percentage of untreated control cultures.

### 2.10. Statistical Analysis

Analysis and graphing of data were performed using Prism 5.0 (GraphPad Software, San Diego, CA, USA). Values are expressed as the mean ± SD. Statistical analysis was performed via analysis of variance (one-way ANOVA) followed by the Student-Newman-Keuls test for significance.* P* < 0.05 was considered to indicate statistical significance.

## 3. Results

### 3.1. DJC Inhibited the Upregulated iNOS and COX2 in GMCs Induced by AGEs

**Figure 1:**
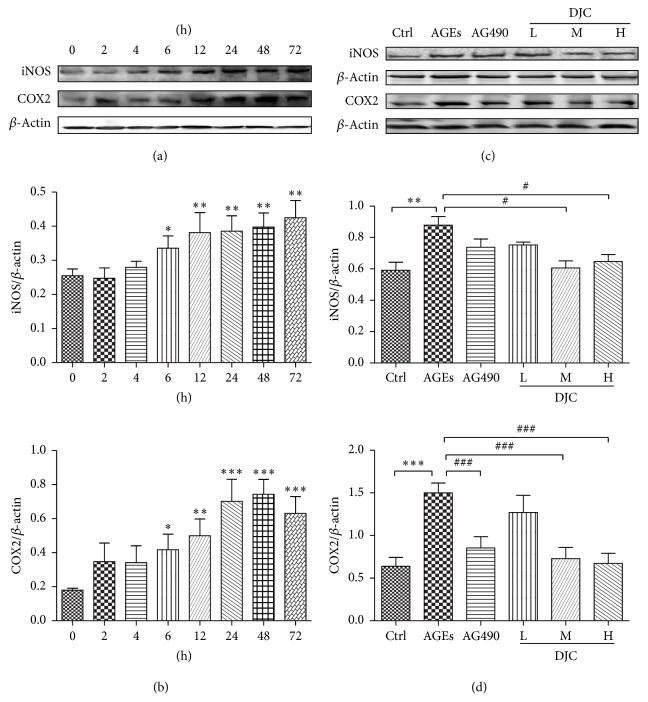
DJC reversed the upregulated iNOS and COX2 in GMCs induced by AGEs. (a) Cells were treated with AGEs (250 *μ*g/ml) for 0, 2, 4, 6, 12, 24, 48, and 72 h. Total cell lysates from GMCs were subjected to analysis for iNOS and COX2 protein levels by western blot. (b) Laser densitometry of the gels showed in (a) and two additional experiments. (c) Total cell lysates from cells treated with AGEs or DJC in the presence of AGEs for 48 h were subjected to analysis for iNOS and COX2 protein levels. (d) Laser densitometry of the gels showed in (c) and two additional experiments. *β*-Actin expression acted as an internal reference. The columns and error bars represent the mean and SD (*n* = 3 per group). ^*∗*^*P* < 0.05, ^*∗∗*^*P* < 0.01, and ^*∗∗∗*^*P* < 0.001 versus Ctrl; ^#^*P* < 0.05, ^##^*P* < 0.01, and ^###^*P* < 0.001 versus AGEs. Similar results were obtained in at least three independent experiments.

GMCs are important intrinsic cells in the renal glomerulus and play an important role in the development of DN. The proliferation of GMCs accompanied by increased extracellular matrix (ECM) accumulation has been proposed to be involved in glomerular basement membrane (GBM) thickening, which eventually results in glomerulosclerosis. Therefore, to investigate the anti-inflammatory actions of DJC and its mechanism, we examined the effect of DJC on the expression of iNOS and COX2 in AGEs-stimulated GMCs. GMCs were stimulated with 250 *μ*g/ml AGEs at different time points, and the cell extracts were assayed using western blot with Abs against either iNOS or COX2 (Figure 1(a)). As shown in Figure 1(b), the elevated COX2 and iNOS were detectable at 6 h, reached the highest level at approximately 48 h, and remained elevated for 72 h in GMCs. Thus, in the current study, GMCs were stimulated with AGEs for 48 h in subsequent experiments.

We then investigated whether DJC could decrease the upregulated COX2 and iNOS in GMCs. GMCs were stimulated with AGEs for 48 h in the absence or presence of different concentrations of DJC. To confirm the involvement of the JAK-STAT pathway, we used the AG490, which is an inhibitor of JAK2 as a positive control (Figure 1(c)). As illustrated in Figure 1(d), AGEs significantly induced the expression of iNOS and COX2 at 48 h, whereas DJC suppressed COX2 and iNOS in a dose-dependent manner. However, AG490 decreased the expression of only COX2, not iNOS, indicating that the upregulated iNOS is not involved in the JAK2-STATs signaling pathway in AGEs-stimulated GMCs.

### 3.2. DJC Suppressed STAT-Responsive Inflammatory Gene Expression

**Figure 2:**
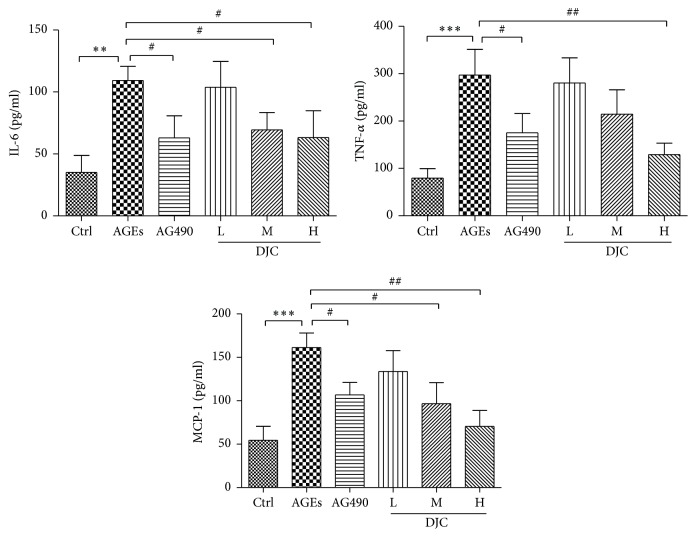
DJC suppressed STAT-responsive inflammatory gene expression. Cells were treated with DJC (0.125, 0.5, and 2 mg/ml) or AG490 (20 *μ*M) in the presence of AGEs (250 *μ*g/ml) for 48 h. The contents of TNF-*α*, IL-6, and MCP-1 in cell culture medium were determined using double-antibody sandwich ELISA. Data are presented as mean ± SD. The columns and error bars represent the mean and SD (*n* = 3 per group). ^*∗*^*P* < 0.05, ^*∗∗*^*P* < 0.01, and ^*∗∗∗*^*P* < 0.001 versus Ctrl; ^#^*P* < 0.05 and ^##^*P* < 0.01 versus AGEs. Similar results were obtained in at least three independent experiments.

Inflammatory cytokines are involved in the development and progression of DN. To further validate the inhibitory effect of DJC on the JAK-STAT signaling pathway, we analyzed the expression profiles of some inflammation-associated genes whose promoters contain the STAT binding sequences. Cells were cultured with AGEs either in the presence or in the absence of DJC for 48 h, and the cytokine concentrations in culture medium were assayed using three commercially available ELISA kits. Following exposure to AGEs for 48 h, GMCs showed higher levels of the inflammatory cytokines IL-6, TNF-*α*, and MCP-1 than those cultured under normal conditions. Treatment with AG490 inhibited the significant increase in TNF-*α*, IL-6, and MCP-1. Moreover, as shown in Figure 2, the three inflammatory cytokines were inhibited by DJC in a concentration-dependent manner.

### 3.3. DJC Inhibited the Activation of STAT1 and STAT3 in AGEs Cultured GMCs

**Figure 3:**
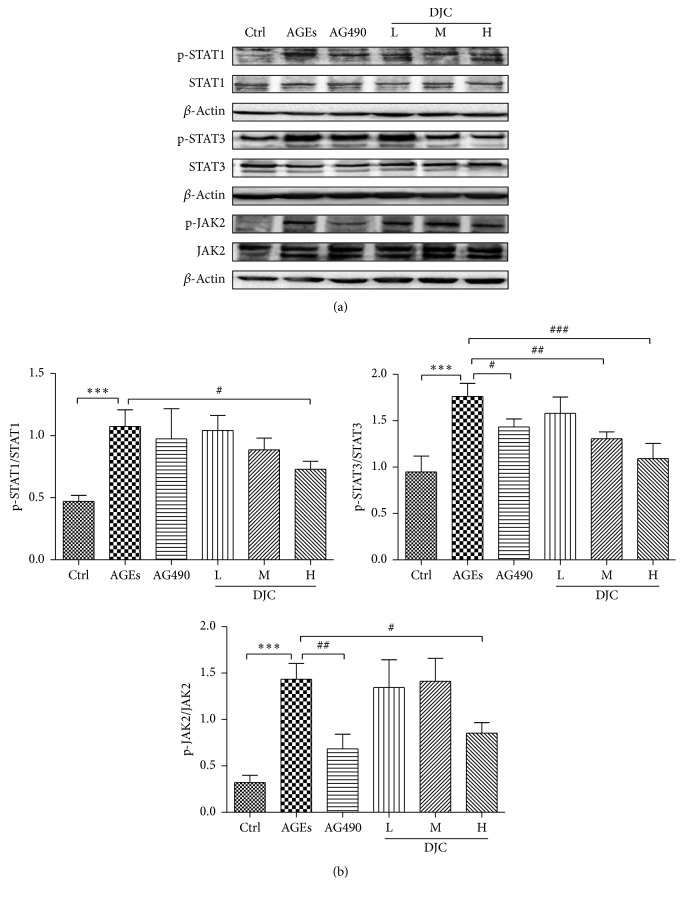
DJC inhibited the activation of JAK2-STAT1/STAT3 pathway in GMCs. (a) Total cell lysates from cells treated with DJC (0.125, 0.5, and 2 mg/ml) or AG490 (20 *μ*M) in the presence of AGEs for 48 h. Proteins were separated by polyacrylamide gels and immunoblotted with anti-phospho-JAK2 (p-JAK2), anti-phospho-STAT1 (p-STAT1), and anti-phospho-STAT3 (p-STAT3) antibodies (upper panels) or antibodies corresponding to the above antibodies (lower panels). This is a representative experiment independently performed three times. (b) Laser densitometry of the gels showed in (a) and two additional phosphorylation experiments. Data are presented as mean ± SD. The columns and error bars represent the mean and SD (*n* = 3 per group). ^*∗*^*P* < 0.05, ^*∗∗*^*P* < 0.01, and ^*∗∗∗*^*P* < 0.001 versus Ctrl; ^#^*P* < 0.05, ^##^*P* < 0.01, and ^###^*P* < 0.001 versus AGEs.

STAT1 and STAT3 have been reported to be important transcription factors for iNOS and COX2 in GMCs. Previous studies [6] have shown that AGEs activated the JAK-STAT pathway, leading to the phosphorylation of STATs. In the current study, GMCs were exposed to AGEs in the absence or presence of DJC for 48 h, and western blot analyses were performed using anti-phosphor-STAT1 (Tyr 701) and STAT3 (Tyr 705) antibodies (Figure 3(a)). As shown in Figure 3(b), the expression levels of the tyrosine phosphorylated STAT1 and STAT3 were significantly increased when cells were treated with AGEs, compared to the nontreated control group (*P* < 0.01). The JAK2 inhibitor AG490 significantly reduced the phosphorylation of STAT1 and STAT3 (*P* < 0.05) without affecting STAT1 or the STAT3 protein levels in GMCs that were cultured in medium containing AGEs.

### 3.4. DJC Inhibited JAK2 Phosphorylation

The phosphorylation of STATs depends on the activation of JAKs. To investigate the signaling mechanism by which DJC modulates the activation of STATs, we examined the effects of DJC on JAK activity. The resulting data are presented in Figures 3(a) and 3(b), and the stimulation of AGEs increased the phosphorylation of JAK2, whereas AG490 and DJC attenuated the phosphorylation of JAK2 in a concentrate-dependent manner but had no effect on the total protein levels of JAK2. These results indicate that DJC inhibits the phosphorylation of STAT1 and STAT3, probably via the inactivation of JAK2 in GMCs. This implies that DJC may exert its therapeutic effects on DN by reducing the AGEs-enhanced activation of JAK2-STAT1/STAT3.

### 3.5. DJC Inhibited the Expression of SOCS3

**Figure 4:**
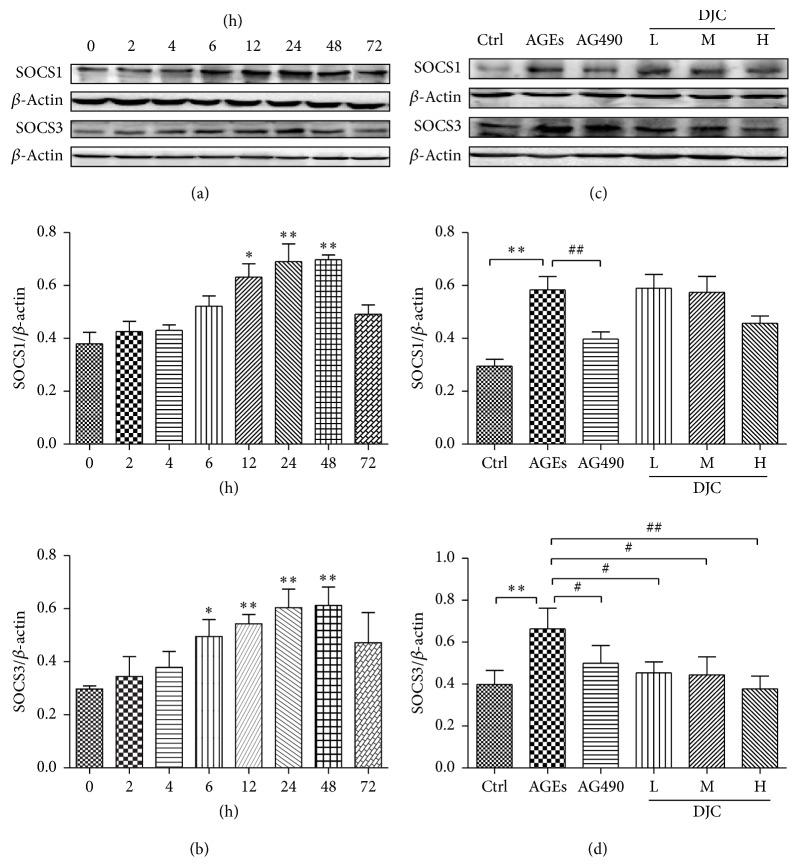
DJC downregulated SOCS expression in GMCs. (a) Cells were treated with AGEs for 0, 2, 4, 6, 12, 24, 48, and 72 h, and then total cell lysates from cells were subjected to analysis for SOCS1 and SOCS3 protein levels. (b) Laser densitometry of the gels showed in (a) and two additional experiments. (c) Total cell lysates from cells treated with AGEs and DJC (0.125, 0.5, and 2 mg/ml) or AG490 (20 *μ*M) in the presence of AGEs for 48 h were subjected to western blot analysis for SOCS1 and SOCS3 protein levels. (d) Laser densitometry of the gels showed in (c) and two additional experiments. *β*-Actin expression acted as an internal reference. The columns and error bars represent the mean and SD (*n* = 3 per group). ^*∗*^*P* < 0.05 and ^*∗∗*^*P* < 0.01 versus Ctrl; ^#^*P* < 0.05 and ^##^*P* < 0.01 versus AGEs. Similar results were obtained in at least three independent experiments.

SOCS proteins have been identified as crucial regulators for the negative regulation of the JAK/STAT pathway [2, 3]. To investigate the mechanism by which DJC inhibits AGEs-induced JAK2-STAT1/STAT3 activation in GMCs, we further examined the effects of DJC on AGEs-mediated SOCS1 and SOCS3 proteins expression. We first examined the effects of AGEs on SOCS1 and SCOS3 expression at different time points (Figure 4(a)). In agreement with previous findings, both SOCS1 and SOCS3 were enhanced by AGEs in time-dependent manners and were detectable at 12 h and 6 h, respectively, but reached the highest level at 24–48 h and then decreased (Figures 4(a) and 4(b)). As depicted in Figures 4(c) and 4(d), DJC and AG490 significantly reversed the AGEs-induced SOCS3 protein expression (*P* < 0.05). However, DJC did not significantly affect the elevated SOCS1 protein levels. These results indicate that the elevation of SOCS1 and SOCS3 proteins expression is involved in the mechanism of activation of JAK2-STAT1/STAT3 pathway by AGEs. DJC negatively regulated AGEs-triggered JAK/STAT signaling pathway.

### 3.6. DJC Inhibited AGEs-Induced ROS and NADPH Oxidase Activity

**Figure 5:**
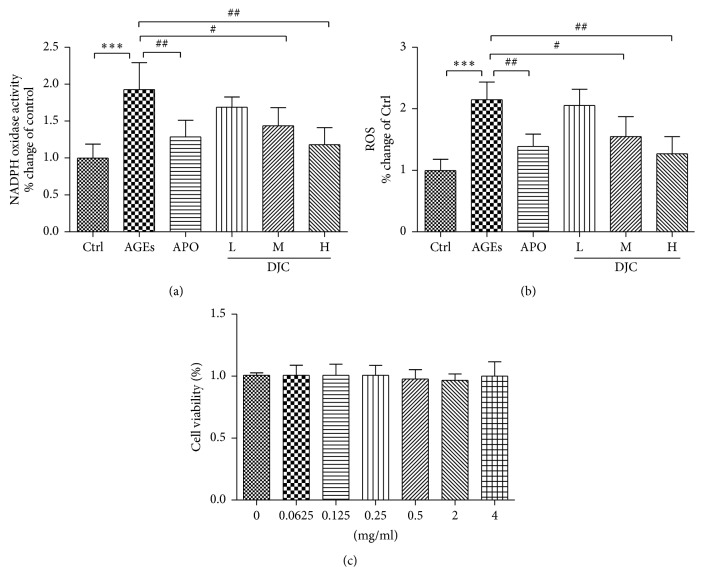
DJC inhibited AGEs-induced ROS and NADPH oxidase activity. Cells were treated with 5% FBS (control), AGEs, and DJC (0.125, 0.5, and 2 mg/ml) or APO (20 *μ*M) in the presence of AGEs for 48 h. The cellular ROS was measured by DCFH-DA (a), and the NADPH oxidase activity was assayed by lucigenin-enhanced chemiluminescence (b). (c) Effects of DJC on cell viability. GMCs were treated with various concentrations of DJC for 48 h. Cell viability was determined by MTT assay. Control in the absence of DJC and AGEs was taken as 100%. The results were expressed as mean ± SD of three independent experiments. The columns and error bars represent the mean and SD (*n* = 4 per group). ^*∗*^*P* < 0.05, ^*∗∗*^*P* < 0.01, and ^*∗∗∗*^*P* < 0.001 versus Ctrl; ^#^*P* < 0.05 and ^##^*P* < 0.01 versus AGEs.

ROS has been considered a central mediator in the progression of diabetic complications. ROS triggers STAT3 tyrosine phosphorylation and nuclear translocation, resulting in the activation of the JAK-STAT signaling pathway [21, 22]. To determine whether the anti-inflammatory effect of DJC is due to its antioxidant effects, GMCs were treated with DJC for 48 h and compared with cells treated with APO (20 *μ*M) as a positive control. APO is known to inhibit JAK/STAT signaling [23, 24] and the expression of inflammatory genes under certain conditions [25, 26]. As illustrated in Figures 5(a) and 5(b), DJC reduced ROS production and NADPH oxidase activity in a dose-dependent manner, thus confirming its antioxidant activity.

### 3.7. Cytotoxicity of DJC

To determine the cytotoxicity of DJC, we investigated its effects on cell viability using the MTT assay. GMCs were incubated with graded concentrations of DJC for 48 h. The results of the MTT assay showed that DJC, even at a high concentration of 4 mg/mL for 48 h, did not affect cell viability (Figure 5(c)), thus demonstrating that DJC is not cytotoxic at the tested concentrations of 0.125–2 mg/ml.

## 4. Discussion

Our previous studies have shown that DJC can reverse STZ-induced DN in rats, reduce urinary protein and urine microalbumin, decrease the serum levels of MCP-1, TGF*β*1, TNF-*α*, CXCL-5, and CXCL-7, inhibit the inflammatory reaction, and improve the microinflammatory state [12–14]. The anti-inflammatory actions of DJC are thought to be attributed to the inhibition of NF-*κ*B activation, which is a mediator involved in cytokine signaling and inflammation. However, no report was available on the effects of DJC on JAK-STAT inflammatory signaling.

The JAK-STAT pathway plays a pivotal role in immune and inflammatory responses and is activated by many cytokines and various pathogenic mediators during DN. Therefore, we tested whether the anti-inflammatory action of DJC is related to the suppression of JAK-STAT activation in GMCs. We examined the effects of DJC on the expression of iNOS and COX2 in GMCs and found that DJC significantly reversed AGEs-inhibited iNOS and COX2, which contain STAT binding sites in their promoter regions. Our finding is in agreement with previous reports [6, 27] and supports our hypothesis that DJC suppresses inflammatory responses via the inhibition of JAK-STAT signaling. In subsequent experiments, we found that treating GMCs with DJC and JAK2 inhibitor AG490 markedly reversed the AGEs-induced JAK2-STAT1/STAT3 activation. Moreover, the ability of DJC to suppress AGEs-induced STAT activation was also verified by the observation that it reversed the increased level of SCOS3 protein induced by AGEs. Taken together, our data support a novel biological function of DJC and suggest a possible molecular mechanism for its action.

AGEs are able to activate JAK/STAT signaling cascade [5, 6], and STATs directly upregulate the transcription of SOCS proteins [28], and thus SOCS proteins are considered as the downstream signal of AGEs activated JAK/STAT cascade. SOCS family members, particularly SOCS1 and SOCS3, inhibit JAK/STAT signaling [29] through several mechanisms, including direct JAK inhibition, STAT binding, and targeting receptor complex and other signaling proteins for proteasomal degradation [30–32]. Therefore, SOCS proteins create part of a classical negative feedback loop [32, 33]. Similar to the previous study [6, 27, 29], we found that SOCS1 and SOCS3 were induced by AGEs from 12 to 48 h in GMCs and then decreased to baseline. Our results indicate that the elevated SOCS1 and SOCS3 proteins are involved in AGEs-stimulated JAK2-STAT1/STAT3 pathway. DJC decreased the enhanced SOCS3 induced by AGEs, which further confirmed that DJC negatively regulates AGEs-induced JAK-STAT signaling pathway. However, we did not find the obvious inhibition of SOCS1 by DJC in this study.

It has been reported that AGEs regulate different transcription factors via activation of JAK/STAT signaling pathway [5, 6]. Huang et al. showed an AGEs-mediated increase in collagen IV production in rat kidney fibroblasts via JAK2-STAT1/STAT3 activity [6]. AGEs have been considered to be among the most toxic substances and are involved in the generation of oxygen free radicals, thereby causing oxidative damage, particularly to the kidney [34, 35]. The formation of AGEs generates extracellular ROS due to the autooxidation of glucose and protein glycation. Extracellular AGEs also bind to RAGE, which is expressed in GMCs and other renal cell types. This initiates intracellular ROS production. Therefore, the accumulation of AGEs induces abnormally high levels of ROS in renal tissue. AGEs have been reported to activate JAK-STAT signaling in mesangial cells, renal tubular cells, and renal interstitial fibroblast cells [6, 7, 36]. The JAK-STAT signaling pathway activation is ROS-dependent. ROS, including hydrogen peroxide (H_2_O_2_) and superoxide anions (O_2_^−^), are potent inducers of various signaling pathways encompassing MAPKs and JAK-STAT pathways. Simon et al. [21] and Gorina et al. [37] reported that JAK2 activation in vitro depends on the presence of ROS. ROS was shown to activate STAT1 and STAT3 and resulted in the nuclear translocation of phosphorylated STATs [21]. Schieffer et al. [38] reported that Ang II stimulated JAK-STAT cascade requires O_2_^−^ anions generated by the NAD(P)H oxidase system in rat aortic smooth muscle (RASM) cells. Ushio-Fukai et al. [39] also reported that Ang II induced a rapid increase in intracellular H_2_O_2_ via NAD(P)H oxidase, which subsequently activated growth-related responses. Similar results have also been reported for PDGF-induced cell proliferation, which was shown to be dependent on H_2_O_2_ [21]. Furthermore, PDGF uses H_2_O_2_ as a second messenger to regulate STAT action in rat fibroblasts [21]. Therefore, the ROS generating system derived from NAD(P)H oxidases is involved in JAK-STAT cascade in response to diverse stimuli, such as Ang II, cytokines, AGEs, and hyperglycemia [38, 40]. APO, the NADPH oxidase and ROS inhibitor, was reported to significantly reduce phosphorylated STAT3 [23, 24] and some inflammatory genes [25, 26]. It is possible that the inhibition of ROS and NADPH oxidase activity may suppress the JAK-STAT signaling cascade [40]. Therefore, we used APO as a positive control in our experiments. Our results showed that DJC reduced the AGEs-induced ROS production and NADPH oxidase activity in a concentration-dependent manner (Figures 5(a) and 5(b)). Therefore, we propose that the inhibition of JAK-STAT signaling possibly occurs via ROS scavenging by DJC.

Following the activation of JAK-STAT signaling pathway, the phosphorylated STATs form dimers that translocate into the nucleus to regulate the transcription of target genes encoding inflammatory mediators such as iNOS and COX2 and inflammatory cytokines. Thus, COX2 and iNOS are involved in renal inflammatory responses in DN. In this study, we found that iNOS was stimulated by AGEs, but AG490 did not reduce the upregulated iNOS, indicating that iNOS expression is not involved in the JAK2-STAT1/STAT3 signaling pathway in AGEs-stimulated GMCs. Previous studies have shown that NF-*κ*B and p38 MAPK pathways are related to AGEs-induced iNOS expression in GMCs [41]. Therefore, we deduce that other signaling pathways may also be responsible for the decreased production of iNOS caused by DJC. In this study, COX2 was stimulated by AGEs and restored by DJC and AG490, suggesting that DJC inhibits AGEs-induced COX2 by blocking the JAK2-STAT1/STAT3 signal and subsequent transcription.

It has been shown that inflammatory cytokines such as IL-6, TNF-*α*, and MCP-1 can be stimulated by AGEs or ROS [42–44]. Thus, to validate the functional importance of the inhibitory activity of DJC on the JAK-STAT inflammatory cascades in GMCs, we examined the expression of inflammatory mediators such as TNF-a, IL-6, and MCP-1 that contain a STAT binding site in their promoter regions. The results showed that AG490 significantly suppressed AGEs-mediated TNF-*α*, IL-6, and MCP-1 and that DJC decreased the production of these inflammatory cytokines in a concentration-dependent manner.

## 5. Conclusions

In conclusion, the present study shows that DJC exerts anti-inflammatory activity by suppressing the AGEs-induced activation of JAK-STATs signaling and through its antioxidant effects. Our results provide new insights into the anti-inflammatory activity of DJC in GMCs. However, further studies are needed to determine the effects of DJC in vivo.
